# A Department of Public Health

**Published:** 1911-07

**Authors:** 


### Abstracts and Selections.

### A Department of Public Health.

In the Senate of the United States.—February 2, 1911. Referred to the Committee on Public Health and National Quarantine.

Amendment prepared by Mr. Owen to his bill (S. 6049) establishing a Department of Public Health, and for other purposes, viz.: strike out Section 8 and insert the following:

"Nothing contained in this bill shall be construed in any manner to authorize the Department of Health to exercise any of the functions exclusively belonging to any of the several States with regard to the health service, unless expressly invited by the proper authorties of such State. No officer of the Department of Health shall be authorized by this act to enter the residence or abode of any person unless invited so to do by the inmates thereof.

"In making examinations for positions in the service of the Department of Health no discrimination whatever shall be made against any applicant on account of any system or school of medicine, but all applications shall be treated alike without regard to the system or school of medicine to which such applicant may have attached himself."

SENATOR OWEN'S STATEMENT.

Senate bill No. 6049, to establish a Department of Health, merely brings into co-ordination the various health activities of the Federal government, putting them under a responsible head whose dignity justifies his being a cabinet officer.

If it were impossible to have a cabinet officer, a director general, independent of other supervision than that of the President, would suffice until this matter were more thoroughly appreciated.

I recently introduced an amendment doing away with Section 8, providing for the establishment of chemical and biological standards—since this was a stumbling block to some—and inserted in lieu of its provisions: first, that no employe of the Department should be discriminated against, in examination for qualification, on the ground of his being attached to any school or system of medicine; second, that no officer of this Department should perform any duty properly belonging to the several States under the constitutional law; and, third, that no officer of this Department should enter any home without the invitation of the inmates thereof.

This removes every point of objection that has been raised against the bill, and leaves it entirely free from blame even by its most hypercritical adversaries.

ROBERT L. OWEN.

Write your Congressman to vote for the Owen bill to establish a National Department of Health.—From the Bulletin of the Committee of One Hundred on National Health, Organ of the American Health League.

---

A DECISION has recently been handed down by the Supreme Court of Minnesota, as mentioned in a recent issue of this journal, which is extremely interesting and may have a far-reaching effect. The cases were those of Delia Keever and Kate Flanagan, each an administratrix against the city of Mankato, their contention being that the municipality was liable for its negligence in its private or corporate capacity, in that it negligently allowed the water supply in its waterworks system to become polluted, causing deaths from typhoid fever. The court held that the city was liable for neglect in its private or corporate capacity and was not exempt because it was performing a governmental function. The *State Journal*, of Parkersburg, W. Va., says with respect to the decision: "Good logic is undoubtedly shown in the Supreme Court's decision, but it is staggering to contemplate what the effect would be in this city if a similar decision were given against Parkersburg of cases of typhoid fever each year, and in practically every case the cause has been traced to the use of the Ohio River water, the city supply. There are several deaths annually from this cause, and time and again the department of health has sounded a warning against the use of the sewage-polluted water for this very reason."—*Lancet-Clinic.*

